# Visualization of Nucleic Acids in Microand Nanometer-Scale Biological Objects Using Analytical Electron Microscopy

**DOI:** 10.32607/actanaturae.27483

**Published:** 2024

**Authors:** O. S. Sokolova, T. S. Trifonova, N. I. Derkacheva, A. V. Moiseenko

**Affiliations:** Lomonosov Moscow State University, Faculty of Biology, Moscow, 119234 Russian Federation; Russian University of Medicine, Department of Biochemistry, Moscow, 127473 Russian Federation

**Keywords:** energy dispersive X-ray spectroscopy, electron energy loss spectroscopy, elemental mapping of phosphorus, bacteriophage, P. aeruginosa, SARS-CoV-2, tick-borne encephalitis virus

## Abstract

Analytical electron microscopy techniques, including energy-dispersive X-ray spectroscopy (EDX) and electron energy-loss spectroscopy (EELS), are employed in materials science and biology to visualize and chemically map diverse elements. This review presents cases of successful identification of nucleic acids in cells and in DNA- and RNA-containing viruses that use the chemical element phosphorus as a marker.

## INTRODUCTION

The precise identification and ultrastructural localization of molecules, organelles, cells, and other biological structures are fundamental in determining their functions. The localization of macromolecules is achieved through immunolabeling techniques on tissue sections, facilitating the detection of specific targets [1]. Cryotomography enables the visualization of the tissue structures [2] and cryo-EM structures of protein macromolecules with atomic resolution [3, 4].

Nobel Prizes awarded for advancements in microscopy highlight the significance of high-resolution molecular imaging, which encompasses the use of green fluorescent protein (GFP) in living cells [5], the circumvention of the diffraction limit through superresolution light fluorescence microscopy [6], and cryoelectron microscopy (cryoEM) [7].

Transmission electron microscopy (TEM) is a common technique used to study the structure of tissues, cells, organelles and protein molecules, which in turn helps to understand the mechanisms underlying the cellular function under normal and pathological conditions. Advancements in electron microscopy and computational power have established TEM as the premier structural biology technique over the past decade. The application of CryoEM allows for the visualization of the three-dimensional architecture and dynamic behavior of a wide array of biological nano- objects at resolutions ranging from 2 to 5 nm to atomic levels [3, 4]. Unlike other structural methods, cryoEM presents several advantages: it is not limited by particle size, the presence of crystals is not necessary, and a small amount of material is used. In addition, cryo-modification of the TEM method permits the visualization of molecules in their native, aqueous environment under near-physiological conditions, which is particularly important for the study of their functional features.

**Fig. 1:**
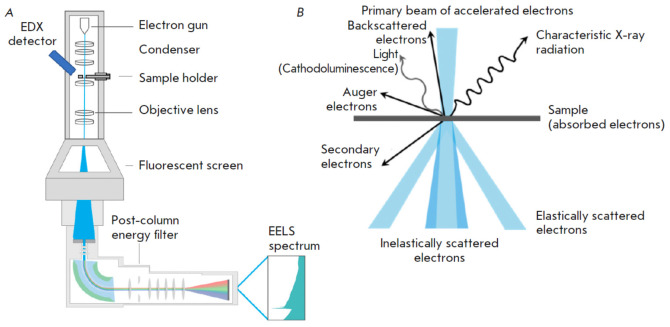
(*A*) The architecture of an electron transmission analytical microscope. (*B*) Visualization of the energy distribution within a thin material following the passage of an accelerated electron beam and the subsequent secondary emissions (generated using BioRender.com)

The underlying principle of transmission electron microscopy is the scattering of an electron beam by a thin section of the material being studied (*Fig. 1A*). Electron-atom collisions within a material give rise to several observable effects such as high-angle elastic scattering, inelastic scattering with concomitant energy loss, the generation of secondary electrons and ionization of target atoms, and characteristic X-ray emission (*Fig. 1B*). The nature of the observed phenomena is determined by factors such as the specific structure of the object under the electron beam, the distribution of the scattering potential, the average atomic number, the thickness of the object, and other parameters. Transmission electron microscopy facilitates the detection of signals that can account for the structure of the object in question.

## ANALYTICAL ELECTRON MICROSCOPY

Biological and materials science research requires not only qualitative but also quantitative analysis, including mapping the elemental composition of microscopic sample areas. For this purpose, scanning and transmission analytical electron microscopes are employed. Analytical TEM detects inelastically scattered electrons. These electrons lost kinetic energy while traversing the sample within the microscope column (*Fig. 1B*).

The analytical electron microscope incorporates specialized detectors facilitating the chemical state analysis of samples via EDX (energy-dispersive X-ray spectrometry) or EELS (electron energy-loss spectrometry). Analytical TEM techniques offer a unique opportunity for acquiring nanometer-resolution elemental compositional data of the investigated specimens [8, 9]. Common analytical TEM methods employed for elemental analysis within biological samples include EDX [10], EELS, and EELS-based elemental mapping by energy-filtering TEM (EFTEM).

## THE ENERGY DISPERSIVE X-RAY SPECTROSCOPY (EDX) METHOD

**Fig. 2:**
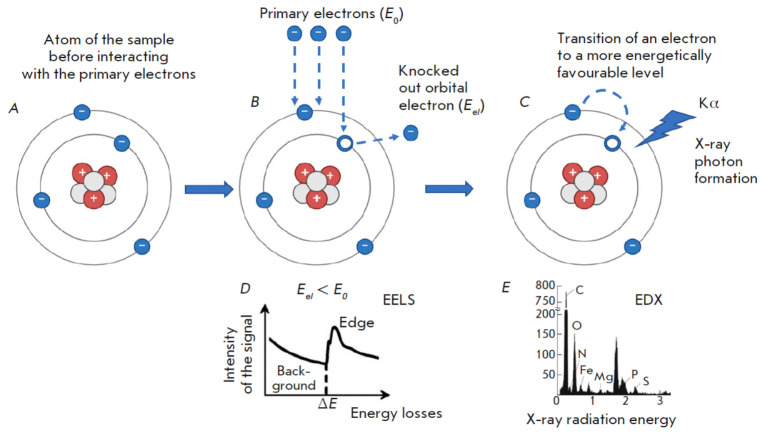
Excitation of the inner shells of a sample atom in a transmission electron microscope column and the resulting EELS and EDX spectra. (*A*) The sample atom before interaction with the primary electron. (*B*) Energy loss detected by EELS.* E*0 – energy of the primary electron before interaction with the sample; *Eel *is the energy of the primary electron after interaction with the sample. (*C*) The generation of the X-ray radiation quantum detected by the EDX method. *Kα *is the X-ray photon generated by the transition of the sample atom from the excited one. (*D*) The electron energy loss spectrum (EELS). (*E*) The energy dispersive spectrum (EDX) (generated using BioRender.com)

The EDX method is based on detecting X-ray photons emitted by samples during electron irradiation (*Fig. 2C*) and measuring their energies. Given the unique, quantized energy values of each atom, X-ray spectra are linear and identifiable to specific elements. The location of peaks on the abscissa axis of a typical EDX spectrum corresponds to the energy values of the X-ray photons absorbed by the detector: the higher the energy, the more to the right the peak is shifted (*Fig. 2E*). The amplitude of each peak is a function of the number of pulses detected on each respective channel.

Elemental analysis using the EDX method applies energy dispersive spectrometers (for example, X-Max, Oxford Instruments, UK). Elemental distribution mapping is achieved through electron energy loss spectrometers, which are typically integrated into transmission electron microscope columns.

Energy-dispersive X-ray spectra from specific areas of the sample are typically recorded using scanning transmission electron microscopy (STEM). In contrast to TEM, the electron beam in STEM is focused using electron optics to form a small probe that scans a thin sample. Biologically significant elements (P, N, O, K, Ca, Mg, Na, Cl, and S) exhibit their most intense X-ray emissions within the 0.15–4 keV energy range (*Fig. 2E*). The EDX method is generally considered a qualitative method, with its primary goal being the identification of a specific Kα peak. Specialized tables and databases facilitate the determination of characteristic X-ray peaks. Automated peak identification software is a standard feature in most X-ray analysis programs.

X-ray quantitative analysis achieves analytical accuracy at the ~1% level, making it possible to compare the content of the element in question in different cells and tissues when normalized to the carbon peak and superimposing the resulting graphs onto each other (see below). It is important to note that discrepancies in quantification may arise from the similar binding energies of specific chemical elements. For example, the Kα peak (2.013 keV) of phosphorus (P) is very close to the M-line (1.914 keV) of osmium (Os), which is commonly used to fix cell membranes. Consequently, differentiating between osmium membrane labeling and phospholipid membrane composition presents a challenge. The use of alternative heavy-metal compounds, for example manganese (distinguished by its unique peak position), is proposed to avoid spectral peak overlapping.

## DETECTION OF PHOSPHORUS ON CELL AND TISSUE SECTIONS USING EDX

Phosphorus, a crucial macronutrient for living organisms, is a constituent of vital compounds, including nucleic acids, ATP (adenosine triphosphate), and phospholipids. It plays a critical role in cellular energy processes [11]. Many species of microalgae and cyanobacteria are characterized by phosphorus storage in the form of intracellular polyphosphate inclusions [12].

Of particular interest is DNA mapping, because it records phosphorus distribution – one atom bound to each nucleic acid base [13]. In recent years, a surge of interest has also been witnessed in the visualization of DNA within nanoparticles and origami structures [14].

**Fig. 3:**
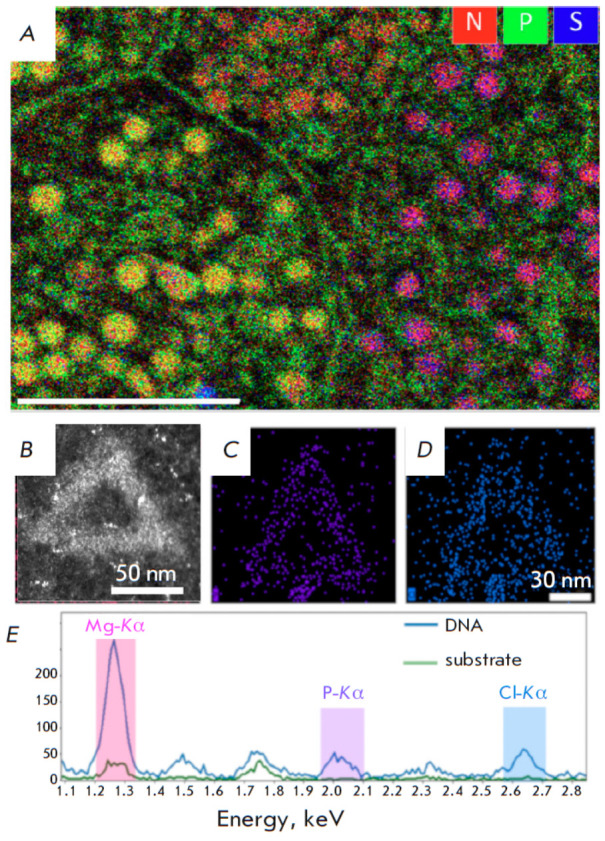
Elemental mapping performed on cellular sections via EDX. (*A*) Rat Langerhans cells islet (reproduced from [16], open source). Overlapping the nitrogen (red), phosphorus (green), and sulfur (blue) compositional maps facilitates the discrimination of membranes and pellets according to their elemental content. The length of the scale bar is 2 μm. The HAADF image of the DNA-origami triangle (*B*). EDX mapping of phosphorus (C) and chlorine (D) distribution. (*E*) Two summed EDX spectra: the solid blue line is summed over the DNA structure while the green line is summed over the background support. Both spectra represent raw data without background subtraction (reproduced from [14], by permission of the authors)

Electron transmission microscopy first allowed researchers to visualize isolated DNA over 75 years ago [15]. A novel circular sputtering technique using heavy metals was developed for the purpose. Uranyl acetate negative staining has been extensively employed for the electron microscopic visualization of DNA and chromatin structures since the 1960s. Nonetheless, analytical TEM is still more frequently employed to identify phosphorus within cellular structures. The qualitative measurement of endogenous elements – like phosphorus in membranes and DNA, nitrogen in polypeptides, and sulfur in methionine- and cysteine-rich proteins ¾ can be conducted pointwise or areawise through the use of EDX and then superimposed on a map. This methodology allowed reasearchers to determine the location of nitrogen- and phosphorus-containing granules in eukaryotic cells (*Fig. 3A*) [16]. Using the same technique the complex elemental composition of vacuolar inclusions of green microalgae were determined [17]. The pphosphorus and other elements were identified in slices of* Drosophila *larval [18], the myelin sheath of human peripheral nerve [19], and in DNA origami (*Fig. 3 B–E*).

**Fig. 4:**
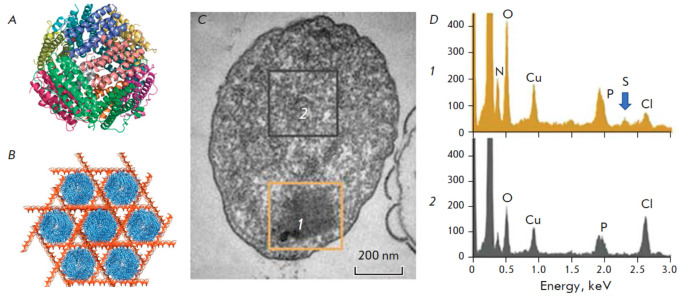
(*A*) The structure of the Dps protein. (*B*) The model of nanocrystalline array during the formation of the DNA-Dps complex based on cryotomography (reproduced from [23], open source); (*C*) The TEM image of a nanocrystalline condensed structure of DNA-Dps in a resting *E. coli *cell starved for 7 months (reproduced from [20], open source): (*1*) nanocrystalline condensation type, (*2*) nucleoid control region. (*D*) The EDX spectra from selected regions *1 *(condensed nucleoid) and 2 (control) in the previous image. The blue arrow indicates the position of the sulfur peak in the spectrum of region 1

Recent studies that have employed the EDX method have elucidated the interaction between DNA and Dps proteins within bacterial cells [20]. Dps, a DNAbinding protein, plays a substantial role in shaping the architecture of the bacterial nucleoid [21]. Dps, a ferritin- like protein, is a dodecamer composed of twelve monomers. Each monomer possesses four alpha-helical subdomains arranged to create a dodecahedron exhibiting tetrahedral symmetry (*Fig. 4A*). Elevated Dps synthesis constitutes a common bacterial response to stress [22]. The cytoplasm of starved cells exhibits two- and three-dimensional crystal lattices, structures formed by Dps molecules interspersed with DNA helices (*Fig. 4B*). The crystallization process safeguards DNA from detrimental environmental factors.

A novel method utilizing analytical electron microscopy was developed to confirm the formation of a DNA-Dps complex. The authors of the method hypothesized that the Kα (2.307 keV) peak of sulfur indicates the existence of the DNA binding protein Dps (each Dps protein contains 48 methionine residues) and that the Kα (2.013 keV) peak corresponds to the phosphorus in DNA. Furthermore, the co-occurrence of both peaks in the EDX spectra was supposed to indicate the formation of a DNA-Dps complex (*Fig. 4D*). The results obtained indicated that in the nanocrystal the bulk of the Dps protein is tightly bound to nucleoid DNA, forming a compact structure, which is consistent with previous studies [23, 24]. No sulfur or phosphorus was detected in the control areas (*Fig. 4D*). The significant copper (Cu) signal detected in all the samples originated from the underlying copper substrate meshes.

**Fig. 5:**
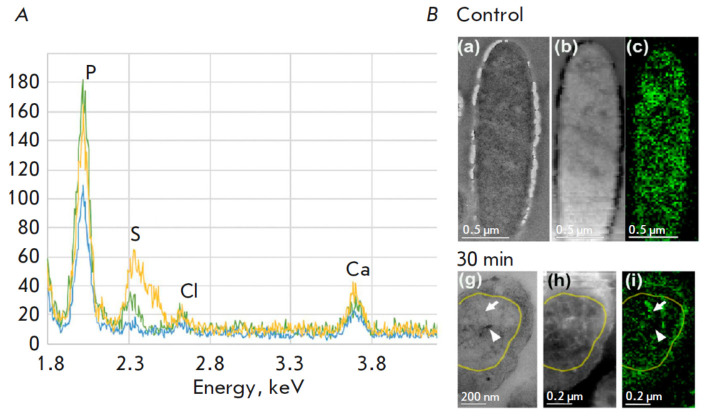
Analysis of the elemental structure of bacteriophage PhiKZ-infected *P. aeruginosa *cells was conducted using analytical microscopy techniques. (*A*) EDX spectra of control cells (blue), and 15 (green) and 30 min (yellow) after infection with PhiKZ phage. The superimposed EDX spectra were normalized to the carbon peak (not shown in graph). The peaks are designated as follows: P for phosphorus, S for sulfur, Cl for chlorine, and Ca for calcium. (*B*) Distribution of phage and bacterial DNA in the control cells and those infected with *P. aeruginosa*. TEM images (a, g), HAADF images (b, h), EELS of phosphorus in the pseudonucleus (c, i). The phosphorus signal (P) is shown after background subtraction and multiple scattering correction, using Fourier deconvolution of the spectra. The arrows indicate DNA, the arrowheads indicate P-free regions, and the yellow line indicates the pseudonuclear boundary (reproduced from [25], open source)

An analogous approach was utilized to analyze the stress response following a bacteriophage infection in *Pseudomonas aeruginosa* [25]. The spectral overlap between the phosphorus Ka peak (2.013 keV) and the osmium (Os) M-line (1.914 keV), a consequence of osmium fixation, was circumvented by contrasting samples with 2% ammonium molybdate. All the EDX spectra were normalized against the carbon peak and then superimposed (*Fig. 5A*).

Post-infection, the phosphorus peak, indicative of the DNA content, demonstrated an increase, seemingly suggesting the contemporaneous presence of both phage and host DNA within the cell. A PCR study demonstrated the persistence of substantial quantities of bacterial DNA 40 min following phage infection [25]. Fifteen minutes after a bacteriophage infection, a small sulfur peak was detected by the authors in the EDX spectral analysis (*Fig. 5A*). This peak increased 30 min after infection, which may be a sign that the bacterial cell responds to stress by increasing the synthesis of the anti-stress protein Dps.

## THE ELECTRON ENERGY LOSS SPECTROSCOPY (EELS) METHOD

The EELS method is based on the detection of the primary signal; namely, the measurement of the energy that is lost by a portion of the electrons that have passed through the sample as a result of the excitation of the sample atoms [8]. Inelastic electron scattering measurements following sample transmission are performed to determine energy loss during spectral acquisition (*Fig. 2B*). Therefore, the EELS approach accounts for all inelastic electron scattering events, such as collective valence electron excitations (plasmons), inner-shell atomic excitations, and the production of bremsstrahlung X-radiation. [26].

When passing through the sample, the primary electron in the transmission electron microscope column interacts with the inner K-shell electron of the sample atom and transfers part of its energy to it (*Fig. 2B*). Consequently, the electron, possessing elevated energy, achieves an excited state. Given that electrons in the ground state occupy all energy levels below the Fermi level, an electron in the excited state can transition only to unoccupied energy levels above the Fermi level. Therefore, when the incident electron undergoes an energy loss surpassing ΔE during its passage through the sample, the probability of transitioning from the K-shell to an energy level above the Fermi level significantly increases. Therefore, the electron energy loss spectrum (the relationship between signal intensity and energy loss) exhibits a sharp peak commencing at ΔE (*Fig. 2D*). Concurrently, this peak exhibits a “tail” within the higher energy range. This shape accounts for the designation of the energy loss spectrum peak as an absorption edge. As the threshold edge energy is unique to each chemical element, the ΔE value in the loss spectrum serves to distinguish the elements in the sample [8], enabling elemental analysis and the monitoring of the chemical bonding state and atomic distances by assessing the intensity of characteristic electron energy losses (*Fig. 2D*). The EELS method is typically employed to analyze the fine structure spectra of elements, thereby elucidating the nature of chemical bonds and the electronic structure of materials.

Elemental analysis via EELS typically employs a post-column energy filter (*Fig. 1A*) (for example, GIF Quantum ER, Gatan, USA). In this case, one can refer to energy-filtered transmission electron microscopy (EFTEM). Given the thickness constraints of this analytical method [26], ultrathin sample preparation is standard practice in EELS elemental analysis. EELS spectra are recorded from selected sample sections in the energy range from 100 to 600 eV in the darkfield scanning mode using a HAADF detector. The aforementioned energy range encompasses the most prominent spectral peaks corresponding to the biologically relevant elements phosphorus, nitrogen, oxygen, and calcium, as observed in electron energy loss spectroscopy.

A recent study utilized EELS mapping to depict the localization of phage DNA in the pseudo nucleus of bacteria following infection by the giant phi- KZ bacteriophage [25]. Analysis of the bacterial DNA distribution within the cytoplasm was performed by overlaying a phosphorus signal with a HAADF image of the cell (*Fig. 5B*). Phosphorus signals were detected in all the cells examined, though their spatial distribution demonstrated temporal dependence on the infection. Within uninfected cells, the cytoplasmic phosphorus signal distribution was uniform, consistent with the diffuse nucleoid location (*Fig. 5B* (c)). Fifteen minutes post-infection, a uniform cytoplasmic distribution of phosphorus (nucleoid position) was observed. Thirty minutes post-infection, the pseudonuclei exhibited a near-spherical morphology and demonstrated centripetal migration, consistent with the results of prior research [27, 28]. A marked alteration in the phosphorus distribution revealed a complex phage DNA network structure within the pseudonucleus (*Fig. 5B* (i)).

## IDENTIFICATION AND MAPPING OF NUCLEIC ACIDS IN VIRUSES

At present, the possibility of mapping nucleic acids on the example of much smaller objects – viruses and bacteriophages – is of particular interest. Prior research has already documented elemental analysis of individual virions [19, 29, 30]. A pioneering study in this area was undertaken in 1980 [19]. Phosphorus was mapped on murine leukemia virus (MuLV) particles embedded in an epoxy resin. The authors successfully documented the phosphorus signal, a constituent of the viral membrane lipids. In 1998, a study on cell cultures infected with transmissible gastroenteritis coronavirus was published [29]. The authors reported recording phosphorus signals from individual viral particles within the cells. Nevertheless, the quality of the provided imagery was insufficient, resulting in ambiguous conclusions. In a later study [30], elemental mapping of whole virions of bacteriophage lambda within films was described. All these experiments were performed using the EFTEM method.

In more recent studies, scanning transmission electron microscopy, combined with energy-filtered electron microscopy (STEM-EELS), has been proposed for elemental mapping [31]. This original technique allows imaging at a lower electron dose than traditional EFTEM. The sensitivity of the method is significantly enhanced by implementing a comprehensive STEMEELS analysis and using a cryogenic sample holder to mitigate radiation-induced damage. The cooling sample holder for TEM can maintain the observed sample at liquid nitrogen temperature, which reduces electron beam damage to the sample and allows the structure to be studied at low temperatures. This aspect is especially critical in the handling of biological samples [32].

STEM-EELS analysis was conducted on a range of viruses containing DNA and RNA. Contrasting the sample with 2% ammonium molybdate instead of uranium acetate proved better in determining with more accuracy the position of the phosphorus absorption peak (absorption limit near 132 eV), since the uranium absorption peak (absorption limit 96 eV) is located near the phosphorus absorption peak and interferes with background subtraction. The molybdenum absorption peak is characterized by an absorption limit near 400 eV.

**Fig. 6:**
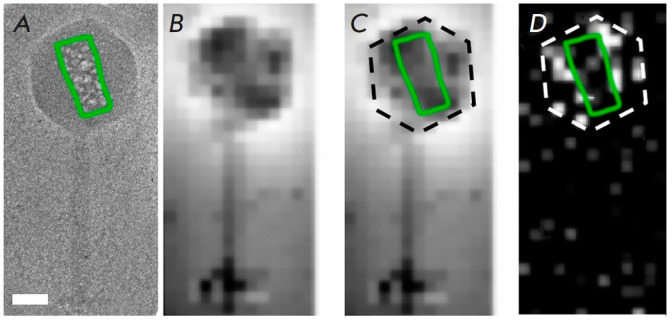
Localization of the inner body in the phiEL bacteriophage, as demonstrated by phosphorus mapping. (*A*) A cryo-electron microscopy image of the phiEL bacteriophage subjected to high-dose electron irradiation. The inner body region is highlighted by a green line. The scale bar measures 50 nanometers. (*B, C*) The HAADF image of the phiEL bacteriophage. A dashed black line denotes the capsid boundary, with the inner body region indicated by a green line (*B*). (*D*) Phosphorus distribution map. The pixel intensities reflect the signal strength of the element in the characteristic electron energy loss spectra. A white dotted line indicates the limits of the capsid, with the inner body region denoted by a green line (reproduced from [31], by permission of the authors)

In testing the STEM-EELS method, the nucleic acid content inside the capsid of the giant phiEL phage was examined. The bacteriophage capsid has a diameter of 145 nm [33], and its genome comprises of 211 base pairs [34]; in other words, it contains 422 thousand phosphorus atoms as part of doublestranded DNA. This study successfully mapped the genomic DNA location within the bacteriophage capsid and confirmed the existence of an internal protein structure around which the DNA is organized (*Fig. 6 C,D*) [31].

**Fig. 7:**
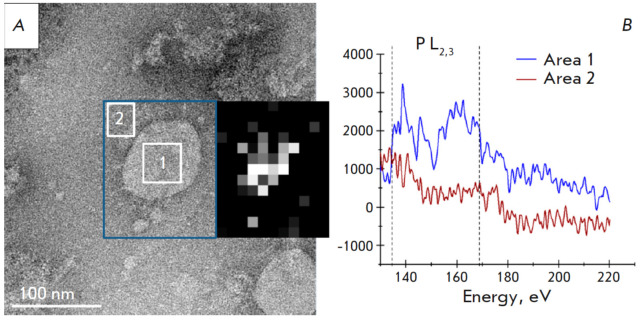
Visualization and elemental analysis of the inactivated SARS-CoV-2 virion. (*A*) Phosphorus distribution map combined with TEM image. (*B*) Plots of EELS spectra obtained inside the virion (region 1 of *Fig*. *7A*) and outside of the virion (region 2 of *Fig*. *7A*) (reproduced from [35], by permission of the authors)

The STEM-EELS method was also used to study the phosphorus content in purified inactivated SARS-CoV-2 particles included in the CoviVac vaccine (produced by the Federal Scientific Center for Research and Development of Immunobiological Preparations named after M.P. Chumakov of the Russian Academy of Sciences) [35]. The SARS-CoV-2 virus has a diameter of around 200 nm and contains a single-stranded RNA genome of approximately 30 base pairs. Thus, the phosphorus concentration within the SARS-CoV-2 capsid is five times lower than that of giant bacteriophages, a difference that remains significant even when accounting for the viral lipid envelope. STEM-EELS analysis confirmed the presence of nucleic acid within the virions. *Figure 7A* shows the SEM image of the virion and the corresponding map of the local phosphorus distribution. The phosphorus signal was recorded only from the interior of the virion, and not from the substrate outside it (*Fig. 7B*). Based on prior findings [36] demonstrating an uneven phosphorus signal distribution within the virion, it is posited that RNA is a more plausible source than the viral lipid envelope.

**Fig. 8:**
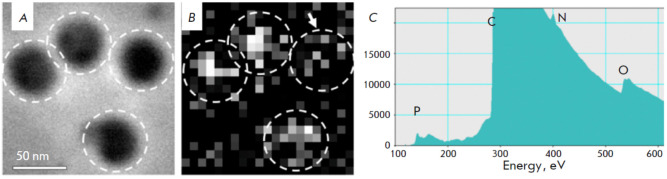
STEM-EELS analysis of TBEV. (*A*) STEM image of TBEV virions. (*B*) Map of the distribution of phosphorus EELS signals within the same sample. The virion boundaries are indicated by white dashed lines, with the arrow pointing to the virion emitting a reduced phosphorus signal. (*C*) EELS spectrum from a single representative virion. Letters P, C, N, and O represent the spectral edge positions for phosphorus, carbon, nitrogen, and oxygen respectively (reproduced from [37], open source)

Finally, the application of STEM-EELS revealed a distribution of significantly less RNA within the purified inactivated tick-borne encephalitis virus (TBEV) virions [37]. TBEV nucleocapsids possess a diameter of 50 nm, while genomic single-stranded RNA comprises approximately 11 base pairs (*Fig. 8A*). Each virion utilized in this experiment demonstrated a phosphorus signal (*Fig. 8B*), with maximal intensity observed in the central region and minimal intensity at the periphery. This indirectly suggests that the signal originated from the phosphorus within RNA. Analogous heterogeneous phosphorus distributions were observed within SARS-CoV-2 virions (*Fig. 7A*) and phiEL bacteriophage capsids (*Fig. 6*). The TBEV virions under investigation exhibited differential signal intensities (*Fig. 8B*). Formaldehyde inactivation [38] likely accounts for the diminished phosphorus signal (*Fig. 8B*) observed in virions, indicating either RNA loss or disruption of the RNA structure.

## CONCLUSIONS

TEM provides high-resolution (nanometer-scale) visualization of the cellular architecture. However, the functional interpretation of macromolecules remains problematic due to the challenges posed by unidentified molecular constituents within the imagery. Combining TEM with EDX permits a high-resolution analysis of endogenous vesicles, diverse tags (including gold or cadmium nanoparticles), and nucleic acids through elemental composition analysis. The application of a cooled sample holder to reduce radiation damage, in conjunction with a comprehensive STEMEELS analysis, allows for the mapping of phosphorus, enabling the determination of nucleic acid location within nanostructures (50–200 nm), which includes inactivated viruses. This methodology has proven effective in visualizing intermolecular interactions and identifying alterations in cellular DNA during a viral infection. Elemental mapping of phosphorus within nano-scale virions using EELS is performed at the detection limit, leading to the aqusition of data with a low signal-to-noise ratio. Nevertheless, the EELS method corroborates the presence of RNA in the majority of the analyzed particles, aligning well with prior research [38].

The application of elemental mapping yields objective biomedical information, as evidenced by the lack of phosphorus signal detection in virus-like vaccine components. Elemental mapping is expected to improve advanced experimental analysis of viruses and virus-like particles, thus establishing analytical electron microscopy as a valuable tool for biomedical product testing.

## Abbreviations

EDX: energy dispersive X-ray spectroscopy
EELS: electron energy loss spectroscopy
TEM: transmission electron microscopy
EFTEM: energy-filtering transmission electron microscopy
STEM: scanning transmission electron microscopy
HAADF: high-angle annular dark-field
TBEV: tickborne encephalitis virus
cryoEM: cryo-electron microscopy
GFP: green fluorescent protein
